# Self-Adapting Short-Range Correlation Functional for Complete Active Space-Based Approximations

**DOI:** 10.1021/acs.jpca.4c03299

**Published:** 2024-08-08

**Authors:** Michał Hapka, Ewa Pastorczak, Katarzyna Pernal

**Affiliations:** † Faculty of Chemistry, University of Warsaw, Warsaw 00-927, Poland; ‡ Institute of Physics, Lodz University of Technology, Lodz 93-005, Poland

## Abstract

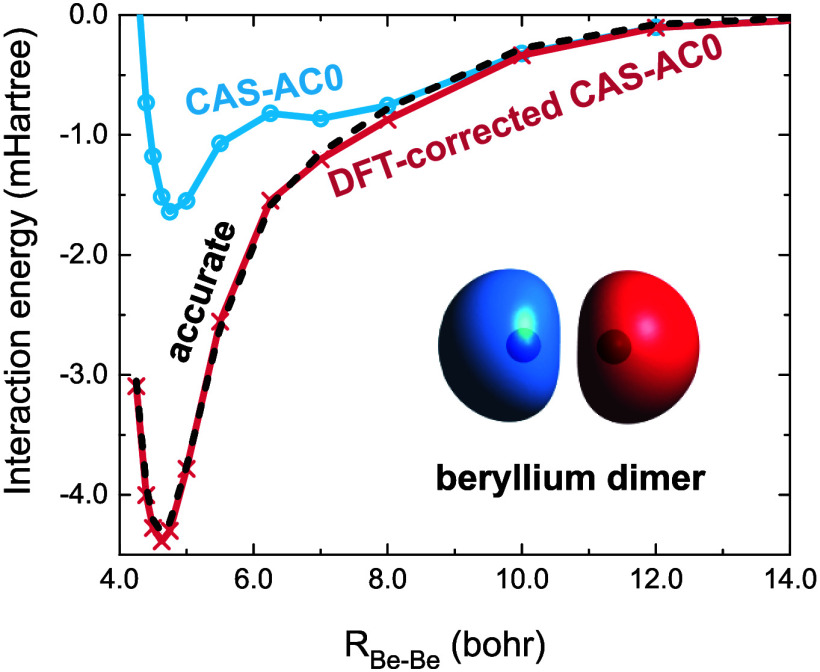

We propose a short-range correlation energy correction tailored for active space wave function models. The correction relies on a short-range multideterminant correlation functional computed with a local range-separation parameter that self-adapts to the underlying wave function. This approach is analogous to that of Giner et al. [*J. Chem. Phys.***2018**, 149, 194301] which addresses the basis set incompleteness error, with the vital distinction that in our protocol the range-separation parameter remains finite in the complete basis set limit, ensuring nonzero short-range correlation. The proposed correlation functional compensates for the missing short-range correlation via two mechanisms: (i) an automatically adapting short-range parameter, which gauges the missing correlation in the electron vicinity, and (ii) the functional’s explicit dependence on the on-top pair density, which reduces short-range correlation in regions where electron correlation is mainly static. We integrate our method into the multireference adiabatic connection theory for CASSCF wave functions. The performance of the introduced CAS-AC0-(c,md) model is verified by calculating potential energy curves for alkaline-earth metal dimers (Be_2_, Mg_2_, Ca_2_) and for the chromium dimer, in all cases obtaining promising results.

## Introduction

Describing the electronic structure of molecules and solids requires models that account for electron correlation effects.^1^ Although it amounts to only a small fraction of the total energy of the system, electron correlation plays a decisive role in the chemical bond formation or van der Waals interactions. Lacking a unique definition, electron correlation energy is conventionally cast as encompassing all effects beyond an independent electron picture. From the perspective of the wave function theory, it is convenient to distinguish between dynamic and static electron correlation. Static correlation appears in multireference cases when the wave function has to be represented as a collection of quasi-degenerate configurations, for example, in a covalent bond-breaking process. The remainder of the correlation energy is called dynamic correlation. It is often associated with a short-range electron interaction^2^ and a buildup of the electron–electron coalescence cusp.^3^ It should be kept in mind, however, that van der Waals interactions, certainly a long-range-electronic phenomenon, also qualify as the dynamic electron correlation effect. For multireference systems, dynamic correlation can be effectively accounted for by perturbation methods, adiabatic-connection-theory-based approximations, or carrying out configuration interaction (CI) expansion of the assumed multireference wave function.

Correlation of electrons can be considered by explicitly partitioning in space the Coulomb interaction into the short- and long-range regimes and applying different approximations to capture short- and long-range correlation energy. This idea has been adopted in multiconfigurational (MC) density functional (DF) theory, lrMC-srDF, proposed by Savin et al.,^4−6^ where the efficiency of density exchange-correlation functional is exploited in the short-range (sr) of electron interaction, and the wave function theory is used in the long-range (lr). The electronic energy follows from the functional of the form:
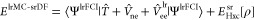
1where T̂ and  denote, respectively, kinetic and electron–nuclei interaction operators, and  stands for a modified electron interaction. By definition,  is such that it is finite at electron coalescence, *r* = 0 and approaches Coulomb interaction in the  limit. Different forms of the operator υ_ee_^lr^ have been considered,^7^ but the most common choice is attenuating the Coulomb operator with the error function whose argument is scaled by a range-separation parameter μ
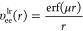
2The short-range Hartree-exchange-correlation energy functional *E*_Hxc_^sr^ is evaluated for an electron density ρ corresponding to a wave function Ψ^lrFCI^. The latter is a full configuration interaction (FCI) function corresponding to a long-range Hamiltonian that includes a modified electron interaction operator and a short-range local potential, . The sr potential ensures that the resulting electron density is exact.

The lrMC-srDF approach offers a number of advantages over wave function and density functional theories alone. Unlike pure DFT, it is capable of targeting multireference systems,^8^ including excited states.^9^ Compared to the wave function formulation, lrMC-srDF exhibits faster convergence with both the basis set size and the active space size. Nevertheless, certain generic limitations persist in the available implementations of lrMC-srDF. The existing short-range exchange-correlation (xc) functionals to a large extent inherit fractional spin and charge errors of the underlying full-range functionals.^10^ Another problem, which is particularly acute when noncovalent interactions play a role, is that correlation effects in the long-range regime are recovered via a wave function approach. This may necessitate long CI expansions, even if selected CI schemes are employed.

To mitigate the dependence of the total energy on the approximate sr exchange-correlation functional, Toulouse and coworkers^11,12^ proposed to include full Coulomb and exchange energies in the wave function part of the functional. In this way, DFT is responsible for capturing only the remaining small fraction of the correlation energy. The energy functional takes the form
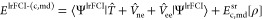
3where the multideterminantal correlation functional  is rigorously defined.^11^ The functional vanishes in the μ → ∞ limit
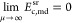
4when the lrFCI wave function tends to the fully correlated FCI wave function corresponding to a Coulomb interaction. Notice that the wave function Ψ^lrFCI^ is obtained using the long-range Hamiltonian  but the energy in eq 3 is computed with the Coulomb operator . Recently, Ferte et al.^12^ have developed an approximate functional depending locally on the electron density and on the on-top pair density, which interpolates between a PBE correlation functional at μ = 0 and the exact known asymptotic in the μ → ∞ limit. The results obtained for the dissociation curves of the diatomic molecules have been promising, but it was due to employing a highly correlated wave function (selected CI), which is not affordable for larger systems. One limitation of the *E*^lrFCI-(c,md)^ model is its dependence on the range-separation parameter μ, akin to the lrMC-srDF approach. The specific choice of μ = 0.5 bohr^−1^ proposed in ref (12) is not universally applicable. From a computational standpoint, a shared drawback of lrFCI-(c,md) and lrMC-srDF is the necessity to handle two sets of electron integrals.

In this work, we adopt the ideas from lrMC-srDF theory in the wave function approach. Specifically, we devise a scheme to correct the recently proposed CAS-AC0 method^13,14^ for the short-range correlation energy. Our approach avoids computation of the near-FCI wave function and requires only conventional, full-range two-electron integrals.

In CAS-AC0, the CAS self-consistent field (CASSCF) energy is corrected for the missing dynamic correlation obtained in the multireference adiabatic connection framework^13^
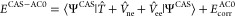
5In practice, the AC0 correction requires solving zeroth-order extended random phase approximation equations^15^ employing one- and two-electron reduced density matrices (1-RDM and 2-RDM, respectively). Since no higher-order density matrices are necessary, the method is computationally more efficient than second-order multireference perturbation theories. Despite its overall good accuracy,^14,16−18^ as a consequence of employing random phase approximation, CAS-AC0 is deficient in capturing short-range correlation.^19,20^ Here, we develop a short-range correlation correction for CAS-AC0 which automatically adapts to the amount of correlation already included in CAS-AC0. The performance of the proposed correction will be illustrated using the example of the dissociation curves of metal dimers: Be_2_, Mg_2_, Ca_2_ and Cr_2_.

## Theory

Recently, Giner et al. developed a density-based basis set correction (DBBSC) method and proposed a functional whose role is to cure the slow basis-set convergence of wave function theories.^21^ For a given wave function model, the functional compensates for the basis set incompleteness error via the local range-separation parameter which automatically adapts to a given basis set. The DFT-based basis-set error correction scheme has been successfully applied for correcting FCI, selected CI, coupled cluster, and GW methods.^22−25^

Exploiting the fact that the basis-set-error-correcting functional essentially captures the short-range correlation energy, which is missing in the wave function method due to a finite basis set and the lack of the electron–electron coalescence cusp, it can be adopted to complement the CAS-AC0 energy. Let us recap the assumptions underlying the DBBSC method introduced in ref (21), as they will pave the way for a short-range correction for CAS-AC0. Recall that the basis-set incompleteness error of the FCI energy in a given basis set  can be formulated as a density functional
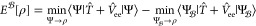
6where  and Ψ are *N*-electron wave functions belonging to the Hilbert space spanned by  and by the complete basis set, respectively. It has been assumed that the density ρ is representable in  and the FCI density obtained in a finite basis set, , is equal to the exact density. Consequently, the basis set correction is obtained as . The exact form of the (basis set dependent) functional  is not known, but it has been associated with the multideterminantal correlation functional , see eq 3, defined as^12^
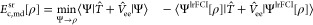
7where Ψ^lrFCI^ is a cuspless wave function. The latter minimizes a model Hamiltonian with the long-range electron interaction and yields a density ρ, namely
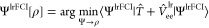
8It has been argued that by a proper construction of the local range separation parameter μ, the short-range behavior of the pair density obtained from the resulting wave function Ψ^lrFCI^ would parallel that of . Therefore, the accuracy of the proposed approximation  depends both on the adopted models for μ and the functional .

Consider the electron-pair density function  of the CAS-AC0 model consisting of the CAS pair density  and the correlation pair density function 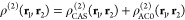
9where  is constructed under the condition that it yields the correlation energy AC0, ∫∫d**r**_1_d. Assuming that CAS-AC0 misses the major part of the short-range correlation energy, it follows that ρ^(2)^ is deficient in the vicinity of the electron coalescence point, . This way, it resembles the pair density corresponding to an electron cuspless wave function Ψ^lrFCI^. We can formally introduce an energy correction for CAS-AC0 as a density functional
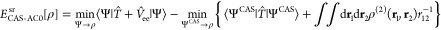
10where Ψ is a wave function belonging to an unrestricted Hilbert space, Ψ^CAS^ is a CAS wave function and the pair density defined in eq 9 includes the AC0 correlation function depending on 1- and 2-RDMs from CAS wave function. (One should keep in mind that even though CAS-AC0 could be formulated as a variational method, in practice the energy is found in a post-CAS manner.) In the spirit of the DBBSC method, we seek for a cuspless wave function Ψ^lrFCI^ which would allow for mapping the functional  onto  given in eq 7. Thus, the key assumption in the construction of the short-range functional for CAS-AC0 is the existence of a range-separation parameter μ, such that the total electronic energy corresponding to the cuspless wave function Ψ^lrFCI^ agrees with that of CAS-AC0, i.e., 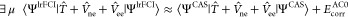
11and the electron densities corresponding to lrFCI and CAS wave functions coincide and are equal to the exact density. By comparing the right-hand sides of eqs 7and 10 computed for the exact density and employing eq 11, it is evident that the  functional is complementary to both lrFCI and CAS-AC0 models and it can be associated with .

In the rest of this section, we closely follow the work of Giner et al.^21^ in constructing the effective μ, which automatically adapts to CAS-AC0 and allows one to compute the electronic energy as a sum of the CAS-AC0 energy and a correlation functional  contribution, namely
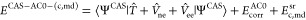
12Using the orbital representation of the spin-free two-electron reduced density matrix, Γ, the pair density reads
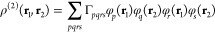
13where *pqrs* denote general orbital indices. The pair density function at coalescence is a local function, known as the on-top pair density, and it will be denoted as Π(r)
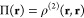
14Following the steps of Giner et al.,^21^ we assume that the key idea in finding the range separation parameter that satisfies the mapping in eq 11 is a construction of the effective electron–electron interaction *w*(**r**_1_,**r**_2_), which (i) yields the same electron repulsion energy as the Coulomb operator  when operating on ρ^(2)^(**r**_1_,**r**_2_)
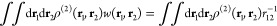
15and (ii) does not have a singularity at r_1_ → r_2_
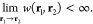
16Since  is finite at electron coalescence, it can be mapped on the long-range electron interaction given in eq 2, by requiring that both functions coincide at coalescence:
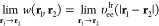
17For the lr interaction given by the error function, , the limit at electron coalescence reads . The  limit combined with the mapping condition of eq 17 allows one to calculate the range-separation parameter μ locally as
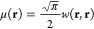
18The particular choice of the effective interaction function is analogous to the one introduced in ref (21)
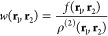
19where the function *f*(**r**_1_,**r**_2_) involves a partial contraction of two-electron integrals with 2-RDM
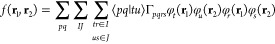
20The ⟨*pq*|*rs*⟩ integrals (a physicist notation is used) are computed with the Coulomb operator. Indices *I*,*J* denote the specific subset of the full set of CAS orbitals, i.e., inactive (doubly occupied), active (fractionally occupied), and virtual (unoccupied in CAS wave function).

First, we notice that the function *w*(**r**_1_,**r**_2_) defined by eqs 19,20 satisfies the requirements given in eqs 15,16. Second, we highlight important differences between the definitions of the function *f*(**r**_1_,**r**_2_) in eq 20 and the one given in ref (21). The form of the *f*(**r**_1_,**r**_2_) function introduced in ref (21) is similar to eq 20, but the summation over the *r* and *s* indices was not restricted. The goal of Giner and coauthors^21^ was to construct a basis set correction based on the  functional which vanishes in the complete basis set (CBS) limit, see eq 4. This imposed a condition that in the CBS limit the *f*(**r**_1_,**r**_2_) function tends to infinity at electron coalescence, r_1_ → r_2_, which leads to infinite μ(**r**) [cf. eqs 18,19]. The singularity of *f*(**r**_1_,**r**_2_) at particle coalescence was achieved by leaving the summation with respect to indices *r* and *s* unconstrained in eq 20 and relying on the CBS limit: . Our goal is different. The correlation correction , see eq 12, should add the missing correlation energy even at CBS, except for a special case when all orbitals are active in CAS, meaning that CAS is equivalent to a FCI wave function. Only in that special case, our constructions of the function *f*(**r**_1_,**r**_2_) and, consequently, of the local function μ(**r**) are identical with those in ref (21). We conclude that μ(**r**) tends to infinity at each **r** only in the FCI, CBS limits:
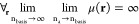
21where n_basis_ and n_a_ stand for the number of basis set functions and the number of active orbitals, respectively. μ(**r**) will be finite otherwise, and it will tend to increase with the expansion of the basis set and/or the expansion of the active space.

The local range-separation function μ(**r**), found according to the procedure outlined in eqs 18−20, is used in the correlation functional , see eq 12. Recall, that it is the same functional which complements the energy with the full-range Coulomb electron interaction corresponding to the Ψ^lrFCI^ wave function, see eq 3. The functional has been rigorously defined,^11^ but its exact form is not known. Local approximations
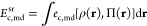
22depending on electron density ρ(**r**) and on-top pair density Π(**r**) have been proposed^11,26^ by exploiting the exact large-μ asymptotic expansion.^27,28^ We will use the recent construction of Ferte et al.^12^ in which the energy density e_c,md_ depending locally on μ is given as
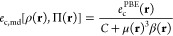
23
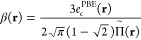
24
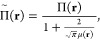
25where *e*_c_^PBE^ is the PBE correlation energy density.^29^ The functional  would have a correct asymptotic behavior in the limit of large μ, if the exact (corresponding to a Coulomb operator) on-top pair density were used for .^27,28^ Since it is not available, in practice, the extrapolation formula given in eq 25 is applied with the available approximate on-top function Π(**r**). We will use the CAS on-top pair density for Π(**r**) in eq 25, as it satisfies the condition that , see eq 21. Notice that in the original proposal for  of Ferte et al.^12^ the parameter *C* was set to 1 to satisfy locally the small-μ condition, .

To summarize, the short-range correlation energy correction added on top of the CAS-AC0 energy, eq 12, is found by computing a local range-separation parameter from eqs 18−20, followed by its application in the local correlation functional defined by eqs 22−24. The most time-consuming step in the whole procedure is the evaluation of the local effective interaction *w*(**r**,**r**) due to a many-fold summation in eq 20. Since its direct implementation would be prohibitively expensive, in the Appendix we show how to reduce the computational cost by exploiting the Cholesky decomposition of the Coulomb integrals.

## Computational Details

All CASSCF calculations were performed in a developer version of the Molpro^30^ program. The CASSCF, FCI and lrFCI results for BH were obtained in Dalton.^31^ Both CAS-AC0 and CAS-AC0-(c,md) methods were implemented in the GammCor^32^ code, which takes 1- and 2-RDMs as input. Dunning basis sets^33^ were used throughout: cc-pVTZ (the BH and N_2_ molecules), aug-cc-pV5Z (Be_2_ and Mg_2_) or cc-pV5Z (Ca_2_) for alkali-metal dimers interaction energy curves, and cc-pV5Z-DK^34^ for the chromium dimer. For the BH molecule, the CASSCF active space consisted of 4 electrons in 5 orbitals, CAS(4,5), which include 2s and three 2p boron orbitals and 1s hydrogen orbital. For N_2_, we employed the CAS(10,10) wave function, which has been demonstrated to be the optimal choice for CAS-AC0.^14^ For alkaline-earth metal dimers, CAS(4,8) active spaces including the valence s and p orbitals of both atoms were used. For the chromium dimer, we used the CAS(12,12) reference, i.e., twelve valence electrons in the 3d and 4s Cr atoms shells. For all metal dimers, we use the counterpoise correction of Boys and Bernardi^35^ to remove the basis set superposition error in the interaction energy.

The lrFCI wave function for the BH molecule was obtained by minimizing the functional of eq 1 with the short-range PBE functional.^36^ The lrFCI results along the dissociation path were calculated with different values of μ at each interatomic distance. At each point, we used the averaged value of μ obtained from the independent CASSCF calculation from the formula of ref (23): , where *N* = 6 is the number of electrons, and μ(**r**) is calculated from eqs 18−20.

## Results

### Validation of the  Functional Adapted to the CAS-AC0 Model

**Figure 1:**
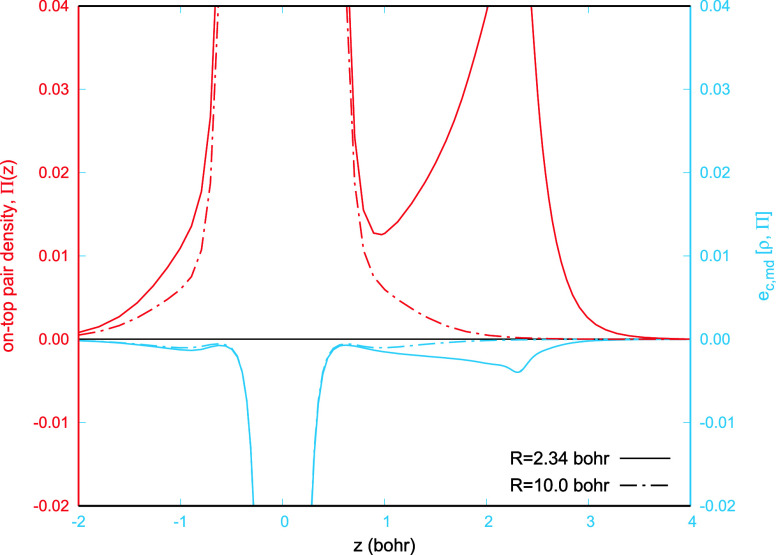
On-top pair density of the BH molecule from CASSCF (red curves), and the local correlation functional *e*_c,md_ defined by eqs 22−25, (black curves), computed at equilibrium geometry and in the dissociation limit. The boron nucleus is located at z = 0 bohr.

The correlation energy functional, defined by eqs 22−24, is adjusted to the wave function theory by scaling the PBE correlation energy density. The scaling explicitly depends on the range-separation parameter μ and on the on-top pair density. As mentioned above, see eq 4, the short-range correlation energy is properly scaled down to zero in the μ → ∞ limit. However, it should also approach zero in the regions where the correlation energy is purely static. In Figure 1, we illustrate this mechanism on the example of the BH dissociation. If the BH bond is stretched, the on-top pair density in the vicinity of the hydrogen atom decreases (the probability of finding two electrons in this region tends to zero) and *e*_c,md_ vanishes. It is worth mentioning that the mechanism of local suppression of the dynamic correlation energy based on the static-correlation-governed value of the on-top pair density has recently been exploited in the CASΠDFT method.^37−40^ In this approach, the correlation correction to the CASSCF energy is obtained via a Π-dependent scaling function multiplying a local correlation functional.

The BH dissociation provides a numerical justification of the central assumption of eq 11, underlying the short-range correlation functional that complements the CAS-AC0 model. We validate the procedure of finding the range-separation parameter μ(**r**) from eqs 18−20 by showing that the lrFCI energy is close to that of CAS-AC0.

**Figure 2:**
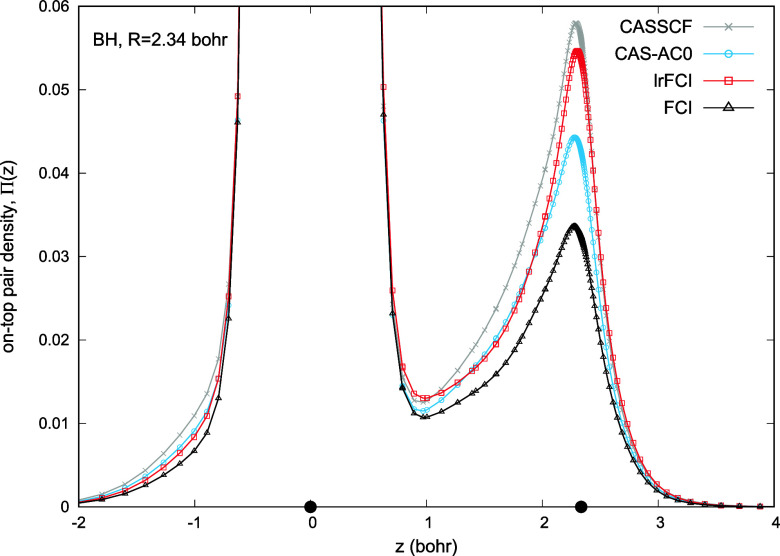
On-top pair densities for BH plotted along the bond axis. Circles indicate positions of the B and H nuclei z= 0 bohr and z= 2.34 bohr, respectively.

Let us first inspect the on-top pair density functions obtained from CASSCF, CAS-AC0, and lrFCI density matrices and compare them with their FCI counterpart. In Figure 2, we observe that the CASSCF on-top pair density has a significantly higher value than the FCI one everywhere along the bond axis, which provides a clear confirmation that the short-range correlation is missing from CASSCF. Accounting for the AC0 correlation correction in the on-top function leads to the CAS-AC0 curve, which is lower than the CASSCF one but, still, remains quite far from FCI. When analyzing the CAS-AC0 pair density, it is important to keep in mind that the underlying CAS-AC0-reconstructed 2-RDM violates N-representability conditions, which could lead to an unphysical behavior of the on-top pair density. For the BH molecule, the lrFCI on-top pair density curve, Figure 2, closely follows the CASSCF and CAS-AC0 curves. This indicates that the μ value found according to the procedure described above leads to a lrFCI wave function exhibiting similar behavior in the vicinity of the electron-cusp as that of CASSCF and CAS-AC0. Using the value of the on-top pair density as a measure of the short-range electron correlation, one can conclude that the lrFCI wave function includes less short-range correlation than its CAS-AC0 counterpart. This is also an indication that the range-separation parameter, found according to the procedure described above and employed in the short-range correlation functional , might lead to a partial double counting of the short-range correlation energy, already accounted for by CAS-AC0.

**Figure 3:**
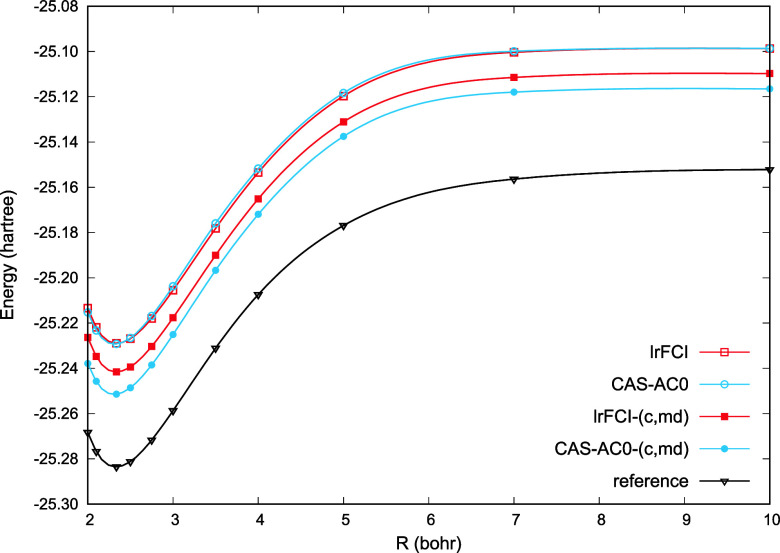
Total energy of BH as a function of interatomic distance. Results of Lie and Clementi^41^ are used as reference.

Second, in Figure 3, we directly verify the assumption of eq 11 by comparing the lrFCI and CAS-AC0 energies. Results from both methods remain in excellent agreement, with deviations of no more than 1.8 mHa along the entire dissociation curve. Adding the correlation energy  to CAS-AC0 and lrFCI energies leads to similar dissociation energy curves, see Figure 3. The quantitative agreement of the CAS-AC0-(c,md) and lrFCI-(c,md) energies validates employing the  functional for CAS-AC0, originally developed for lrFCI. Results in Figure 3 were obtained by setting *C* = 5 in eq 22. We have found that the originally proposed *C* = 1 parameter typically leads to overbinding, which is the case for BH/cc-pVTZ. The dissociation energy predicted by the lrFCI-(c,md) functional amounts to 135.2 mHa and 133.1 mHa for the values of the *C* parameter equal, to 1 and 5, respectively. The accurate value reported by Lie and Clementi^41^ is 131.3 mHa.

Apparently, the local range-separation parameter computed according to the proposed procedure is typically too small, leading to overcorrelation if employed in the approximate complementary functional  with *C* = 1. In the Supporting Information (Tables S1 and S2) we show the energies and the dissociation energies obtained with various values of *C* for the nitrogen dimer in cc-pVTZ and cc-pVQZ basis sets. The results confirm that setting *C* = 1 leads to overcorrelating and overbinding due to double counting of the short-range correlation present in the CAS-AC0 model. This observation necessitates using a larger value of the parameter, and *C* = 5 appears to be the optimal choice.

All results presented in the remaining part of this work were obtained with the *C* parameter of 5. We compare the accuracy of two variants of CAS-AC0-(c,md) method. In the first, the local range-separation parameter, cf. eqs 18−20, is computed based on the 2-RDM from CASSCF wave function. In the second, denoted CAS-AC0-(c,md)’, a correlation correction from AC0 is added to the CASSCF 2-electron reduced density matrix.

### The Nitrogen Molecule, Alkaline-Earth Metal Dimers and the Chromium () Dimer

As a first test case for our models, we calculated dissociation energies of the N_2_ molecule (Table 1). The uncorrected CASSCF energies are compared to CASSCF supplemented with either AC0 or AC0-(c,md) correlation corrections. The dissociation energies were estimated as differences of the total energies computed at *R* = 10 bohr and at the equilibrium interatomic distance, *R*_eq_ = 2.075 bohr. The CAS-AC0 result deviates by 10 mHa from the accurate^41^ value. Adding the  correlation energy computed with the local, self-adapting range-separation parameter, reduces the error to the value below 1 mHa. The CAS-AC0-(c,md) and CAS-AC0-(c,md)’ results differ by less than 1 mHa. The averaged μ obtained at *R*_eq_ in the former method is equal to 1.64 bohr^–1^, and it is only slightly smaller than the value of 1.71 bohr^–1^ corresponding to the latter variant. A smaller range-separation parameter leads to a lower  energy. Consequently, total CAS-AC0-(c,md) energies are by a few mHa lower compared to CAS-AC0-(c,md)’.

**Table 1:** Total energies of N_2_ at equilibrium geometry *R*_eq_ = 2.075 bohr and in the dissociation limit *R*_∞_ = 10 bohr in Hartree, and the dissociation energy *D*_0_ in mHartree in the cc-pVTZ basis set.^33^^a^

	accurate	CASSCF	CAS-AC0	CAS-AC0-(c,md)	CAS-AC0-(c,md)’
*R*_eq_	–109.5340	–109.1674	–109.3768	–109.4516	–109.4493
*R*_∞_	–109.1698	–108.8172	–109.0225	–109.0873	–109.0842
*D*_0_	364.2	350.2	354.3	364.3	365.1

a The accurate values are taken from ref (41).

**Figure 4:**
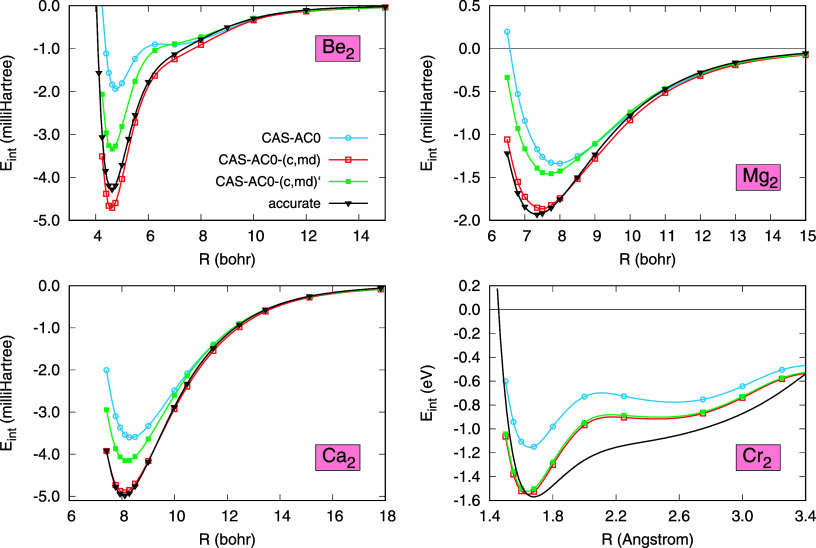
Potential energy curves of Be_2_, Mg_2_, Ca_2_ (energies in milliHartree), and Cr_2_ (energies in eV). Accurate results are taken from ref (44) for Be_2_, ref (45) for Mg_2_, ref (46) for Ca_2_, and ref (47) for Cr_2_ (the revised experimental fit).

Interaction potentials of alkaline-earth metal dimers remain challenging even for modern multireference approaches.^12,42^ Despite their closed-shell character, alkaline-earth atoms exhibit near-degeneracy of *n*s and *n*p orbitals making single-reference description inadequate, which manifests in slow-convergence of the coupled cluster expansion or the Mo̷ller–Plesset perturbation series. At the same time, large polarizabilities of metallic atoms translate into particularly strong dispersion forces,^43^ which require accurate treatment of dynamic correlation at all distances. In Figure 4, we present potential energy curves (PECs) for Be_2_, Mg_2_, and Ca_2_ computed with CAS-AC0 and CAS-AC0-(c,md) methods. For comparison, in the Supporting Information, one can find potential energy curves (PECs) obtained in smaller basis sets of the triple- and quadruple-ζ quality.

In these dimers, static correlation effects are most pronounced in Be_2_. The CAS-AC0 method correctly predicts the position of the minimum, but recovers only 50% of the binding energy with respect to the ref (44). Moreover, the CAS-AC0 curve exhibits a shallow, second minimum at 7.0 bohr. The improvement upon the addition of the short-range correlation via the  functional is noteworthy. The CAS-AC0-(c,md) potential is free of the unphysical minimum and the relative error is reduced to ca. 10% overbinding (−4.719 mHa vs −4.280 mHa at 4.625 bohr). For both Mg_2_ and Ca_2_, the CAS-AC0-(c,md) model performs even better, with relative percent errors of −3% and −2%, respectively (i.e., a slight underbinding). In the case of Mg_2_, the potential becomes repulsive at larger distances compared to the reference,^45^ so that the position of the minimum is shifted by 0.15 bohr. Still, the uncorrected CAS-AC0 underbinds Mg_2_ by more than 30% and places the minimum at 8.0 bohr instead of 7.35 bohr. Although CAS-AC0-(c,md)’ potentials are more accurate than the CAS-AC0 ones, they are clearly inferior to the CAS-AC0-(c,md) results. This most likely reflects a poor quality of the AC0-based correction to 2-RDM for van der Waals systems.

In contrast to the alkaline-earth metal dimers, which are bound by weak intermolecular forces, the ground  state of the chromium dimer exemplifies a covalent bond, with a formal bond order of six as it dissociates into two high-spin  atoms. Similar to Be_2_, both static and dynamic correlation are essential to capture the shape of the potential energy curve, which features a minimum around 1.68 Å and a shoulder in the 2.6 Å region, as derived from photoelectron spectroscopy^48^ and the state-of-the-art *ab initio* investigations.^47,49^ The CAS-AC0 method predicts the global minimum at ca. 1.65 Å and a second flat one is observed in the 4s bonding shoulder region (see Figure 4). The CAS-AC0-(c,md) approach both deepens the global minimum and improves the shape of the PEC in the shoulder region. For Cr_2_, the results from CAS-AC0-(c,md) and the CAS-AC0-(c,md)’ variants stay in close agreement. The CAS-AC0-(c,md) method predicts the equilibrium bond length of ca. 1.64 Å - underestimation compared to the reference value of 1.68 Å. The dissociation energy clearly improves upon addition of short-range correlation: from 1.16 eV at the CAS-AC0 level of theory to 1.55 eV obtained from CAS-AC0-(c,md) calculations, with 1.57 eV being the binding energy from the revised fit of ref (47). Beyond the minimum, the (c,md) potentials deviate more strongly from the experiment, which could result from the lack of 4d orbitals in the active space, e.g., see ref (50). Employing larger active spaces would require going beyond the standard CASSCF model and applying the proposed functional with, e.g., DMRG wave functions, which we plan to explore in future work.

## Conclusions

In this work, we have proposed a short-range correlation energy correction suitable for the active space wave function models. The required amount of the short-range correlation, which must be added to a given wave function method, is accounted for by the short-range multideterminant correlation functional.^12^ The key element in our approach consists in computing the short-range functional with a local range-separation parameter which self-adapts to the wave function theory it is combined with. The idea is similar to that presented by Giner et al.^21^ to cure the basis set incompleteness error. The important difference is that, in our case, the range-separation parameter stays finite in the complete basis set limit, and the short-range correlation is different from zero. The correlation functional accounts for the missing electron correlation via two mechanisms: (i) the automatically adapting μ, whose role is to probe how much correlation is missing in the electron vicinity, and (ii) the explicit dependence of the functional on the on-top pair density. The latter effectively leads to a decrease of the short-range correlation in the regions of space where the on-top pair density vanishes and the correlation becomes purely static.

The proposed method has been combined with the CAS-AC0 approach, where CASSCF captures mostly the static correlation, while the AC0 term adds dynamic correlation. The CAS-AC0-(c,md) model corrects CAS-AC0 for the missing short-range dynamic correlation effects. The new approach has been analyzed and validated based on the dissociating BH and N_2_ molecules.

We have assessed the performance of CAS-AC0-(c,md) by computing potential energy curves for the challenging alkaline-earth metal dimers (Be_2_, Mg_2_, Ca_2_), and the chromium dimer. Excellent accuracy has been obtained with a CAS-AC0-(c,md) variant in which the range-separation parameter is constructed based on 2-RDM from the CASSCF wave function. If the 2-RDM includes a contribution from the AC0 approximation [the CAS-AC0-(c,md)’ variant], the overall performance is similar or slightly worse. The latter could result from the violation of the N-representability by the CAS-AC0 2-RDM. Taking into account that computing the AC0 contribution to 2-RDM significantly increases the cost (see the Appendix), we recommend the CAS-AC0-(c,md) method.

As a final remark, we emphasize that the proposed short-range correlation correction protocol can be combined with approaches other than CAS-AC0, for example, multireference second-order perturbation methods developed for CASSCF or DMRG. Our protocol is computationally efficient; in the recommended variant it scales with the second power of the number of occupied orbitals, with the first power in the number of Cholesky vectors, and the first power in the grid size. Promising future applications include noncovalently bound systems with non-negligible static correlation effects and extending the method to excited states.

## Appendix: Implementation of the  Function

Following the representation of the electron-pair density in eq 9, we divide the *f*(**r**_1_,**r**_2_) function, eq 20, into two parts: *f*(**r**_1_,**r**_2_)=*f*_CAS_(**r**_1_,**r**_2_)+*f*_AC0_(r_1_,r_2_). In the following, the subsets of inactive, active, and virtual orbitals will be denoted as i, a, and v, respectively. Assume natural orbital representation;  denotes a set of natural occupation numbers, so that , with *N* being the number of electrons. Introduce decomposition of two-electron integrals: , where vectors *L* result from Cholesky decomposition of the AO Coulomb matrix and subsequent transformation to the natural-orbital representation. Restricting the summation over orbital indices, see eq 20, and exploiting the block structure of the spin-summed 2-RDM obtained from a CAS wave function
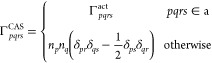
26the *f*_CAS_ function at electron coalescence, r_1_=r_2_, can now be expressed via Cholesky-decomposed integrals
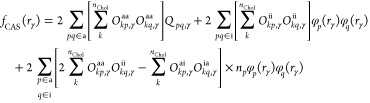
27where *r*_γ_ refers to the -th DFT grid point. The intermediate matrices are defined as
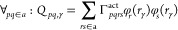
28
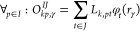
29with *IJ* denoting aa, ii, ai, or ia pairs of orbital subsets. The most computationally expensive steps in the CAS-AC0-(c,md) energy functional, that relies on eq 27, are the evaluation of the *Q* matrix, which scales as , and the evaluation of the *f*_CAS_ formula with roughly the  scaling.

To compute , we define the correlated 2-RDM, denoted , via the AC0 correlation energy formula, so that the condition  is satisfied, where *pqrs* indices run over all orbital basis functions. The actual form of  can be extracted from eq (46) in ref (14). Note that the correlated 2-RDM defined in this way is not *N*-representable and violates symmetry rules. The working expression for the correlated part of the *f* function at coalescence, given for the γ-th grid point, reads
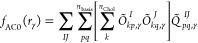
30and
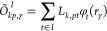
31
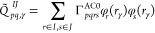
32Computing the *f*_AC0_ formula, eq 30, is the bottleneck of the CAS-AC0-(c,md)’ model which scales as , with the required number of Cholesky vectors *n*_Chol_ ranging from 6 to 8 times the orbital basis set size.

## Data Availability

The raw data are available in the Zenodo repository at 10.5281/zenodo.12565739.

## Supporting Information Available

Total energies and dissociation energies of nitrogen molecule calculated in triple- and quadruple-ζ Dunning basis sets for different values of the *C* parameter. Potential energy curves of Be_2_, Mg_2_, and Ca_2_ computed in triple- and quadruple-ζ Dunning basis sets (PDF)
