# Supplementary material for: Self-Adapting Short-Range Correlation Functional for Complete Active Space-Based Approximations
Source: J Phys Chem A. 2024 Aug 8;128(33):7013–22. doi: 10.1021/acs.jpca.4c03299 (PMC11345816; doi:10.1021/acs.jpca.4c03299)
Supplement: Supplementary file 1 — jp4c03299_si_001.pdf [file jp4c03299_si_001.pdf]

Supporting Information for
"Self-adapting short-range correlation functional for complete active space-based approximations"

Michał Hapka,[1] Ewa Pastorczak,[2] and Katarzyna Pernal[2]

[1]*Faculty of Chemistry, University of Warsaw,*
ul. L. Pasteura 1, 02-093 Warsaw, Poland
[2]*Institute of Physics, Lodz University of Technology,*
ul. Wolczanska 217/221, 93-005 Lodz, Poland

TABLE S1: Total energies of N_2 at equilibrium geometry $R_{\rm eq} = 2.075$ bohr and in the dissociation limit $R_\infty = 10$ bohr in Hartree, and the dissociation energy D_0 in mHartree in the cc-pVTZ basis set [1]. The accurate values are taken from Ref. 2.

		CAS-AC0-(c,md)			CAS-AC0-(c,md)'		
	accurate	$C=1$	$C=5$	$C=10$	$C=1$	$C=5$	$C=10$
$R_{\rm eq}$	−109.5340	−109.6123	−109.4516	−109.4177	−109.5964	−109.4493	−109.4169
R_∞	−109.1698	−109.2211	−109.0873	−109.0583	−109.2026	−109.0842	−109.0572
D_0	364.2	391.2	364.3	359.4	393.7	365.1	359.7

TABLE S2: Total energies of N_2 at equilibrium geometry $R_{\rm eq} = 2.075$ bohr and in the dissociation limit $R_\infty = 10$ bohr in Hartree, and the dissociation energy D_0 in mHartree in the cc-pVQZ basis set [1]. The accurate values are taken from Ref. 2.

		CAS-AC0-(c,md)			CAS-AC0-(c,md)'		
	accurate	$C=1$	$C=5$	$C=10$	$C=1$	$C=5$	$C=10$
$R_{\rm eq}$	−109.5340	−109.6741	−109.5141	−109.4803	−109.6550	−109.5113	−109.4794
R_∞	−109.1698	−109.2753	−109.1492	−109.1209	−109.2529	−109.1451	−109.1195
D_0	364.2	398.8	364.9	359.4	402.1	366.2	359.9

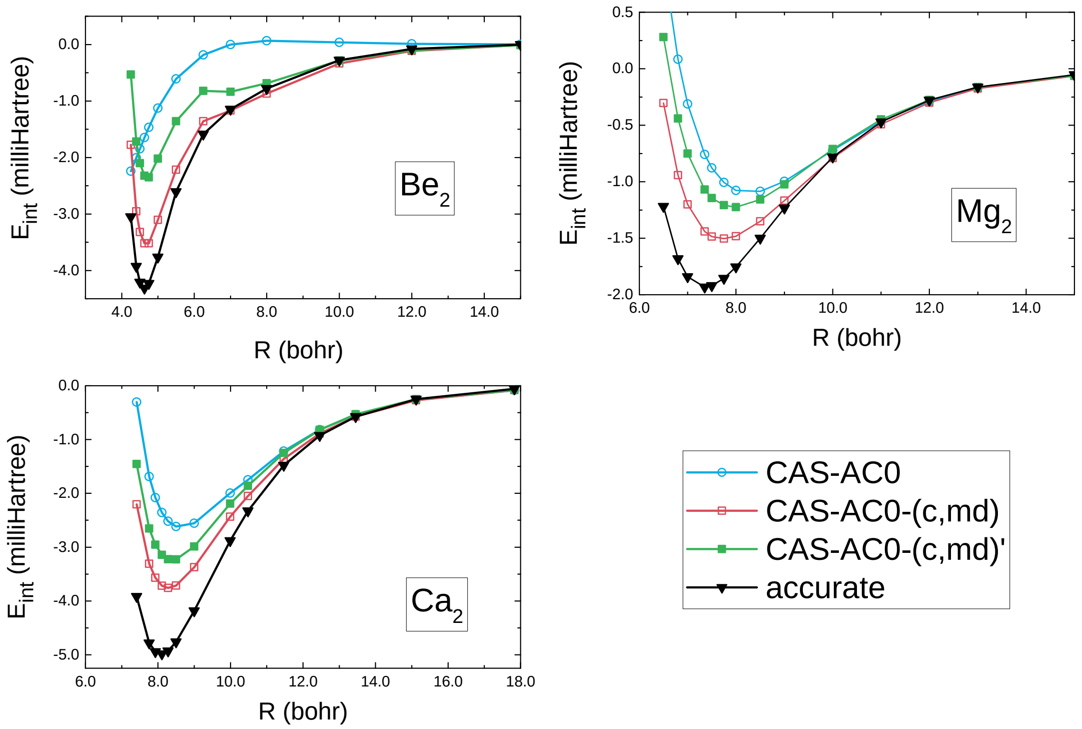

FIG. S1: Potential energy curves (energies in milliHartree) calculated in the aug-cc-pVTZ (Be_2, Mg_2) or cc-pVTZ (Ca_2) basis sets. Accurate results are taken from Ref. 3 for Be_2, Ref. 4 for Mg_2 and Ref. 5 for Ca_2.

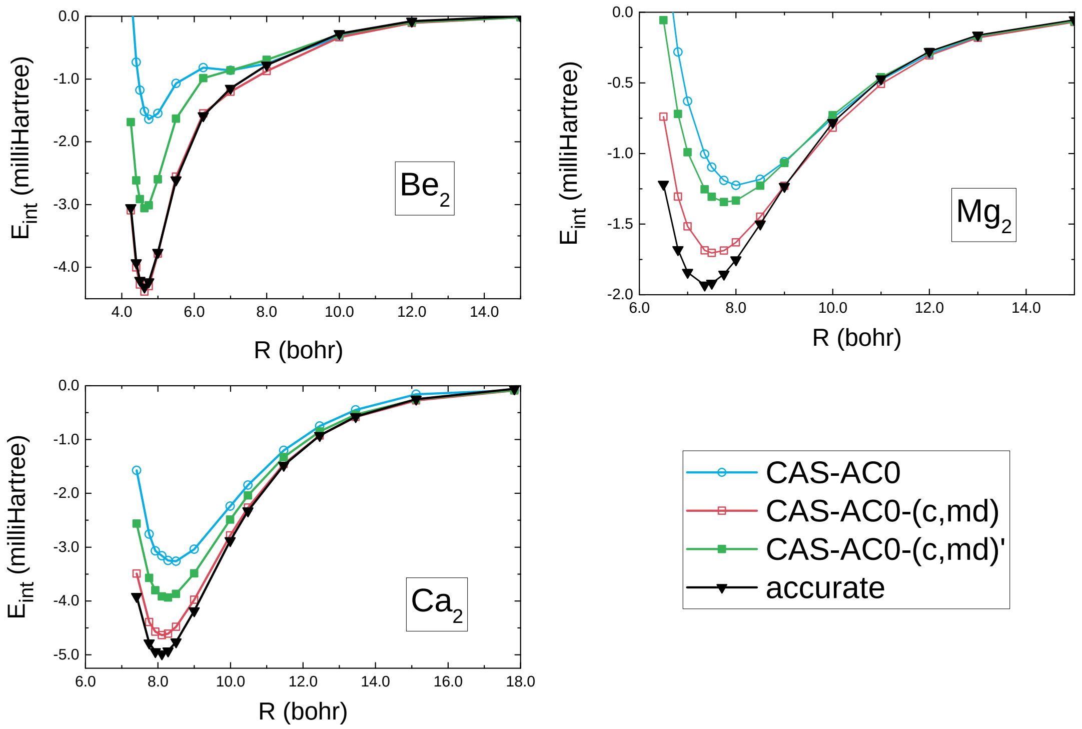

FIG. S2: Potential energy curves (energies in milliHartree) calculated in the aug-cc-pVQZ (Be_2, Mg_2) or cc-pVQZ (Ca_2) basis sets. Accurate results are taken from Ref. 3 for Be_2, Ref. 4 for Mg_2 and Ref. 5 for Ca_2.

[1] T. H. Dunning Jr, J. Chem. Phys. **90**, 1007 (1989).

[2] G. C. Lie and E. Clementi, J. Chem. Phys. **60**, 1288 (1974).

[3] M. Lesiuk, M. Przybytek, J. G. Balcerzak, M. Musiał, and R. Moszynski, J. Chem. Theory Comput. **15**, 2470 (2019).

[4] W. J. Balfour and A. E. Douglas, Can. J. Phys. **48**, 901 (1970).

[5] O. Allard, A. Pashov, H. Knöckel, and E. Tiemann, Phys. Rev. A **66**, 042503 (2002).
